# Clinical outcomes in Caroli disease and Caroli syndrome: a longitudinal observational cohort study

**DOI:** 10.1038/s41598-026-42855-8

**Published:** 2026-03-26

**Authors:** Amr Shaaban Hanafy, Eslam Kamal Fahmy, Rania Naguib, Moaz Abulfaraj, Ahmed F. Omar, Hend Naguib, Mohamed Mahmoud Abdelrahman, Hany A. Elkattawy

**Affiliations:** 1 Internal Medicine Department, Gastroenterology and Hepatology Division, Zagazig University, Zagazig, Egypt; 2 Department of Physiology, College of Medicine, Northern Border University (NBU), Arar, 91431 Saudi Arabia; 3 Department of Internal Medicine, College of Medicine, Princess Nourah bint Abdulrahman University, P.O. Box 84428, Riyadh, 11671 Saudi Arabia; 4 Department of Surgery, Faculty of Medicine, King Abdulaziz University, Jeddah, Saudi Arabia; 5 Department of Hepatology, Gastroenterology & Infectious Diseases, Faculty of Medicine, Zagazig University, Zagazig, Egypt; 6 Internal Medicine Department, Hepatology Unit, Faculty of Medicine, Alexandria University, Alexandria, Egypt; 7 Department of Anaesthesia and Critical Care, King Abdulaziz University Hospital, King Abdulaziz University, Jeddah, Saudi Arabia; 8 Department of Anesthesia and surgical intensive care and pain management, Mansoura Faculty of Medicine, Mansoura University, Mansoura, Egypt; 9 Department of Basic Medical Sciences, College of Medicine, AlMaarefa University, Diriyah, Riyadh 13713 Saudi Arabia; 10 Research Centre, Deanship of Scientific Research and Post-Graduate Studies, AlMaarefa University, Diriyah, Riyadh 13713 Saudi Arabia

**Keywords:** Caroli’s disease, Caroli’s syndrome, Cholangitis, Portal hypertension, Cholangiocarcinoma, Cancer, Diseases, Gastroenterology, Oncology

## Abstract

Caroli’s disease (CD) and Caroli’s syndrome (CS) are rare congenital fibropolycystic liver disorders. While CS is known to be associated with more severe hepatic complications than isolated CD, direct longitudinal comparisons between the two entities remain limited due to their rarity. This single-center, retrospective, longitudinal observational cohort study descriptively compared clinical features, complications, and long-term outcomes in 19 patients diagnosed with CD (*n* = 7) or CS (*n* = 12) between November 2015 and December 2022. Diagnoses were confirmed by magnetic resonance cholangiopancreatography. Patients underwent standardized clinical, laboratory, endoscopic, and imaging assessments during follow-up. Outcomes included recurrent cholangitis, portal hypertension–related complications, hepatic decompensation, cholangiocarcinoma, and all-cause mortality. Statistical analyses were descriptive and exploratory. Patients with CS exhibited more frequent portal hypertension, lower platelet counts, higher bilirubin and international normalized ratio levels, and lower serum albumin compared with patients with CD. All cases of esophageal varices, hepatic decompensation, cholangiocarcinoma (*n* = 3), and death (*n* = 3) occurred exclusively in the CS group. Exploratory analyses demonstrated monotonic associations between adverse outcomes and higher aspartate aminotransferase levels, increased frequency of cholangitis episodes, thrombocytopenia, and elevated CA 19 − 9 levels. Kaplan–Meier analysis showed shorter median survival in CS compared with CD (45.0 vs. 59.0 months; log-rank *p* = 0.020), which should be interpreted descriptively given the limited sample size. In this descriptive longitudinal cohort, Caroli’s syndrome was associated with a more severe clinical course and poorer outcomes than isolated Caroli’s disease, consistent with existing literature. These findings provide detailed comparative outcome data and highlight clinically relevant patterns that may inform surveillance strategies and guide future multicentre, hypothesis-driven studies.

**Graphical Abstract:**

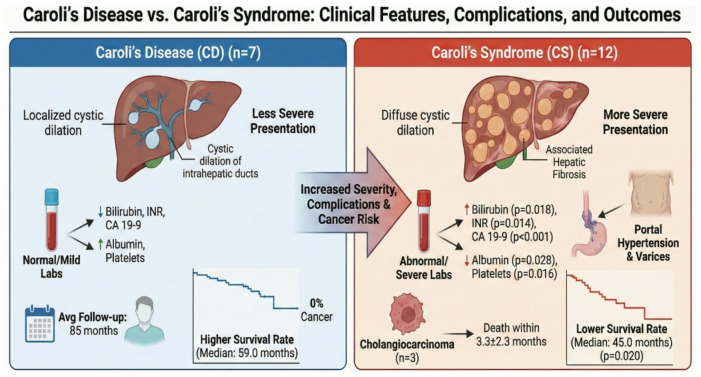

**Supplementary Information:**

The online version contains supplementary material available at 10.1038/s41598-026-42855-8.

## Introduction

Caroli’s disease (CD) is an infrequent congenital disorder characterised by segmental, non-obstructive saccular dilation of the intrahepatic bile ducts. It is categorised into two distinct forms: the uncomplicated form, known as Caroli’s disease (type I), and the more complex variant referred to as Caroli’s syndrome (CS or type II), which is associated with congenital hepatic fibrosis and frequently with renal cystic diseases such as autosomal recessive polycystic kidney disease (ARPKD) or autosomal dominant polycystic kidney disease (ADPKD)^1,2^.

Both congenital dilatation (CD) and congenital stenosis (CS) are components of the spectrum of fibropolycystic disease. These conditions are frequently associated with mutations in the PKHD1 gene, located on chromosome 6p12, which encodes fibrocystin/polyductin—a protein expressed in bile ducts, renal tubules, and pancreatic ducts^3,4^. Such mutations interfere with the normal remodelling of the ductal plate during embryogenesis, leading to malformed intrahepatic bile ducts and periportal fibrosis^5^. Clinically, Caroli’s disease manifests through recurrent episodes of cholangitis, abdominal pain, and hepatobiliary complications. In cases of Caroli’s syndrome, progressive fibrosis may result in portal hypertension and upper gastrointestinal bleeding, particularly from oesophageal varices^6,7^. Imaging modalities are integral to diagnosis, with hallmark features including the “central dot sign,” which is observed on ultrasonography and MRCP. This sign, characterized by a central hyperechoic dot within a hypoechoic saccule, is a specific and sensitive marker for Caroli’s disease and syndrome, facilitating its precise diagnosis^8^.

Although the clinical severity of Caroli’s syndrome compared with isolated Caroli’s disease has been previously recognized, direct comparative studies with long-term follow-up remain limited due to the rarity of both conditions. Most available data derive from case reports or heterogeneous series without standardized longitudinal assessment; the natural history of these diseases remains poorly defined due to their rarity and limited long-term follow-up data. This scarcity of longitudinal evidence complicates clinical management, as it hinders accurate prediction of the timing and frequency of complications such as recurrent cholangitis, portal hypertension, variceal bleeding, and cholangiocarcinoma. Consequently, clinicians face challenges in optimally planning surveillance, timing surgical interventions, and implementing individualized risk-stratified strategies. By providing detailed, long-term observational data from a well-characterized cohort, the present study aims to refine current understanding of disease progression, complications, and outcomes in CD and CS, thereby informing clinical decision-making and guiding future multicenter and molecular studies.

## Patients and methods

This was a single-center, retrospective, longitudinal observational cohort study designed to describe and compare clinical outcomes in patients with Caroli’s disease and Caroli’s syndrome. Patients evaluated between November 2015 and December 2022 were included. The study was approved by the Institutional Review Board of Zagazig University [ZU-IRB # 85-28-24], and written informed consent was obtained from all participants in accordance with the Declaration of Helsinki.

### Patient selection

A total of 19 consecutive patients diagnosed with Caroli’s disease or Caroli’s syndrome were included. Diagnosis was established based on characteristic radiological findings on magnetic resonance cholangiopancreatography (MRCP), demonstrating segmental, non-obstructive dilatation of intrahepatic bile ducts in continuity with the biliary tree. Patients were classified into:

Caroli’s disease (CD): isolated intrahepatic bile duct dilatation without evidence of congenital hepatic fibrosis (*n* = 7).

Caroli’s syndrome (CS): Caroli’s disease associated with features of congenital hepatic fibrosis and/or portal hypertension (*n* = 12).

Patients were excluded if they had prior liver transplantation, chronic viral hepatitis (HBV or HCV, autoimmune liver disease, non-alcoholic fatty liver disease, chronic alcohol use, other congenital hepatic cystic disorders, incomplete clinical records or refusal to participate.

### Clinical and laboratory assessment

At baseline and during follow-up, patients underwent standardized clinical evaluation, including documentation of abdominal pain, fever and recurrent cholangitis episodes, jaundice, gastrointestinal bleeding, and signs of hepatic decompensation.

Laboratory investigations included a complete blood count to assess for thrombocytopenia, which can signal portal hypertension; liver function tests (AST, ALT, alkaline phosphatase, gamma-glutamyl transferase, total bilirubin, albumin); a coagulation profile; and renal function tests. The neutrophil-to-lymphocyte ratio (NLR) was calculated from complete blood counts as an inflammatory marker, given its reported association with severe acute cholangitis and septic shock. Serum CA 19 − 9 levels were measured during follow-up as part of routine clinical surveillance. To exclude alternative aetiologies, serological testing for viral hepatitis, autoimmune liver disease, and parasitic infections (fascioliasis, ascariasis, hydatid disease) was performed in all patients.

### Abdominal ultrasonography

Abdominal ultrasonography was performed to characterize hepatic morphology, identify disease-related complications, and support the radiologic diagnosis of Caroli’s disease and Caroli’s syndrome. The presence of ascites and ultrasonographic features suggestive of chronic liver disease, such as coarse hepatic echotexture, nodular liver surface, and reduced liver size, was documented. Portal hypertension was assessed using established sonographic criteria, including the presence of portal venous collaterals, a portal vein diameter > 13 mm, splenic enlargement (splenic diameter > 130 mm), and/or ascites^10^.

Characteristic segmental intrahepatic bile duct dilatation was recorded, frequently accompanied by intraductal calculi. Additional ultrasonographic findings, including cholelithiasis, pancreatic cystic lesions, and renal cystic abnormalities, were documented when present, reflecting the fibropolycystic disease spectrum commonly associated with Caroli’s syndrome. In patients with suspected malignancy, ultrasonography demonstrated nonspecific features such as intraductal or mass-forming lesions, biliary obstruction, or compression of adjacent structures; these findings prompted further evaluation with cross-sectional imaging and serum tumour markers, as clinically indicated.

### Magnetic resonance cholangiopancreatography (MRCP)

Magnetic resonance cholangiopancreatography (MRCP) was used to characterize the distribution and morphology of intrahepatic biliary abnormalities. MRCP demonstrated segmental dilatation of intrahepatic bile ducts, frequently accompanied by hepatic cysts and, in some cases, intrahepatic abscesses, which are recognized complications within the Caroli disease spectrum. Widespread intrahepatic bile duct dilatation in continuity with the biliary tree was recorded as a key radiologic feature supporting the diagnosis. Characteristic MRCP findings included a peripheral “funnel-shaped” configuration of the intrahepatic bile ducts, with saccular and/or fusiform dilatations distributed throughout the liver. These imaging patterns helped differentiate Caroli’s disease and Caroli’s syndrome from other biliary disorders. In contrast, localized intrahepatic bile duct dilatation without associated cystic or fibrotic changes was interpreted cautiously, as such findings may reflect isolated primary intrahepatic lithiasis rather than true Caroli’s disease^11^.

### Upper GI endoscopy

Upper gastrointestinal endoscopy was performed when clinically indicated to evaluate the presence of portal hypertension–related complications. Esophageal varices were identified and graded according to the Paquet classification system, which categorizes varices from Grade I to Grade IV based on size and degree of luminal protrusion^12^.

### Outcome measures

Outcome measures were descriptively defined to characterize the clinical course and complications associated with Caroli’s disease and Caroli’s syndrome during longitudinal follow-up. Primary outcomes included the occurrence and frequency of disease manifestations, such as fever, right upper quadrant abdominal pain, vomiting, jaundice, and recurrent episodes of cholangitis.

Major clinical complications documented during follow-up included:

i. Portal hypertension–related gastrointestinal bleeding, including oesophageal variceal haemorrhage;
ii. Laboratory abnormalities reflecting hepatic dysfunction, including elevations in aspartate aminotransferase (AST), alanine aminotransferase (ALT), total bilirubin, and international normalized ratio (INR);
iii. All-cause mortality, with specific documentation of cases developing cholangiocarcinoma, diagnosed based on characteristic imaging findings and supported by additional diagnostic procedures, including histopathology when available.

### Secondary outcomes

Secondary outcomes were assessed to provide a broader descriptive evaluation of disease evolution and included:

i. Development of hepatic decompensation, including hepatic encephalopathy;
ii. Radiological disease progression, defined as increased intrahepatic bile duct dilatation, appearance of new cystic lesions, or imaging features suggestive of progressive hepatic fibrosis;
iii. Extrahepatic manifestations, including cystic involvement of the kidneys or pancreas.

### Follow-up duration

Patients were followed longitudinally from November 2015 to December 2022 through regular clinical visits and medical record review. The median follow-up duration was 73 months, permitting descriptive assessment of both short- and long-term clinical outcomes.

### Statistical analysis

Statistical analyses were performed using IBM SPSS Statistics version 25.0 (IBM Corp., Armonk, NY, USA). Given the small sample size and the non-normal distribution of several variables, continuous data are presented as the median with interquartile range (IQR). Comparisons between groups were conducted using the Mann–Whitney U test for continuous variables and Fisher’s exact test for categorical variables, which are suitable for small sample sizes.

Exploratory associations between selected clinical variables and adverse outcomes, including cholangiocarcinoma and all-cause mortality, were examined using Spearman’s rank correlation coefficient. No regression or predictive modelling analyses were performed to avoid overfitting and unstable estimates, given the limited number of observations and outcome events.

To reduce the risk of type I error associated with multiple testing, a Bonferroni correction was applied, and adjusted p-values are reported where appropriate. For descriptive purposes, a two-sided p-value < 0.05 was considered statistically significant. All results should be interpreted as hypothesis-generating rather than confirmatory or predictive.

### Sample size

Caroli’s disease and Caroli’s syndrome are rare conditions, and the sample size in this study reflects the number of patients evaluated at a single tertiary referral center over the study period. The cohort size allows for detailed descriptive characterization of clinical features, complications, and longitudinal course. However, the limited number of patients precludes definitive statistical inference, subgroup analyses, or predictive modeling. All studies and comparisons are therefore intended solely for descriptive and hypothesis-generating purposes.

## Results

A total of 19 patients were enrolled between November 2015 and December 2022, including 7 patients with Caroli’s disease (CD) and 12 patients with Caroli’s syndrome (CS). Patient recruitment occurred between December 2015 and May 2018, after which all patients were followed longitudinally until the end of the study period.

The baseline demographic and clinical characteristics of patients with Caroli’s disease (CD) and Caroli’s syndrome (CS) are shown in Table 1. There was no statistically significant difference in age between the two groups (*p* = 0.374), and sex distribution was comparable (*p* = 0.608). Abdominal pain was present in all patients. Fever was significantly more frequent in the CS group (91.7%) compared with the CD group (42.9%), reaching statistical significance (*p* = 0.038). Jaundice was observed more commonly among patients with CS (66.7%) than among those with CD (14.3%); however, this difference did not reach statistical significance (*p* = 0.147). Gastrointestinal bleeding occurred exclusively in the CS group (33.3%), although the difference compared with CD patients was not statistically significant (*p* = 0.106).

**Table 1:** Baseline clinical characteristics of patients with Caroli’s disease and Caroli’s syndrome.

Variable	Caroli’s disease (*n* = 7)	Caroli’s syndrome (*n* = 12)	*p* value
Age (years), median (IQR)	33 (31–35)	38 (29–44)	0.374
Male sex, n (%)	6 (85.7)	8 (66.7)	0.608
Abdominal pain, n (%)	7 (100)	12 (100)	–
Fever, n (%)	3 (42.9)	11 (91.7)	**0.038**
Jaundice, n (%)	1 (14.3)	8 (66.7)	0.147
Gastrointestinal bleeding, n (%)	0 (0)	4 (33.3)	0.106
Recurrent cholangitis, n (%)	7 (100)	12 (100)	–

Statistical tests: Mann–Whitney U test for continuous variables; Fisher’s exact test for categorical variables.

Laboratory parameters reflecting hepatic function and disease severity are shown in Table 2. Patients with CS exhibited significantly lower platelet counts compared with those with CD (median 120 vs. 213 × 10³/µL; *p* = 0.018). Serum albumin levels were also considerably lower in the CS group (median 3.3 vs. 4.1 g/dL; *p* = 0.031). In contrast, markers of cholestasis and hepatic dysfunction were significantly higher among CS patients, including total bilirubin (median 2.03 vs. 1.30 mg/dL; *p* = 0.020) and INR (median 1.54 vs. 1.10; *p* = 0.016). Serum CA 19 − 9 levels were markedly elevated in the CS group compared with the CD group (median 40.5 vs. 15 U/mL; *p* = 0.009). These findings indicate a significantly more impaired biochemical profile in patients with CS. The neutrophil-to-lymphocyte ratio (NLR) was considerably higher in patients with Caroli’s syndrome compared with those with Caroli’s disease (median 3.8 [IQR 2.9–5.1] vs. 2.1 [IQR 1.7–2.4], *p* = 0.012). This finding indicates a greater systemic inflammatory burden in patients with Caroli’s syndrome and is consistent with the higher frequency of inflammatory complications observed in this group. Given the observational nature of the study and the limited sample size, NLR is presented as a descriptive marker reflecting disease severity rather than a predictive indicator.

**Table 2:** Laboratory findings and disease severity indicators.

Parameter	Caroli’s disease (*n* = 7) Median (IQR)	Caroli’s syndrome (*n* = 12) Median (IQR)	*p* value
Platelet count (×10³/µL)	213 (178–300)	120 (90–197)	**0.018**
Albumin (g/dL)	4.1 (3.8–4.3)	3.3 (3.1–3.7)	**0.031**
Total bilirubin (mg/dL)	1.30 (1.20–1.50)	2.03 (1.60–2.60)	**0.020**
INR	1.10 (1.00–1.17)	1.54 (1.23–1.80)	**0.016**
CA 19 − 9 (U/mL)	15 (12–17)	40.5 (29.3–95.5)	**0.009**
NLR	2.1 (1.7–2.4)	3.8 (2.9–5.1)	**0.012**

Statistical test: Mann–Whitney U test, NLR, neutrophil-to-lymphocyte ratio.

**Table 3:** Outcomes during Follow-up.

Outcome	Caroli’s disease (*n* = 7)	Caroli’s syndrome (*n* = 12)	*p* value
Number of cholangitis episodes, median (IQR)	3 (3–4)	6 (5–7)	**< 0.05**
Portal hypertension, n (%)	0 (0)	9 (75.0)	**0.002**
Esophageal varices, n (%)	0 (0)	5 (41.7)	0.106
Cholangiocarcinoma, n (%)	0 (0)	3 (25.0)	0.263
All-cause mortality, n (%)	0 (0)	3 (25.0)	0.263
Median survival (months)	59	45	**0.02**

Statistical tests: Fisher’s exact test for categorical outcomes, Mann–Whitney U test for episode counts, Kaplan–Meier analysis for survival (descriptive).

Magnetic resonance cholangiopancreatography demonstrated segmental cystic dilatation of the intrahepatic bile ducts in continuity with the biliary tree. MRCP also documented biliary complications, including biliary strictures in two patients and cholangiocarcinoma in three patients. Cholangiocarcinoma manifested either as a focal stricture involving the hepatic duct bifurcation with upstream intrahepatic ductal dilatation (*n* = 1) or as intraductal mass-forming lesions (*n* = 2).

To exclude alternative aetiologies of biliary dilatation, serological testing for fascioliasis, ascariasis, and hydatid disease was performed, with negative results in all patients. Autoimmune markers, including antinuclear antibodies, antimitochondrial antibodies, and anti-liver-kidney microsomal antibodies, were also negative. Screening for viral hepatitis showed negative hepatitis C antibody and hepatitis B surface antigen results in all patients.

Upper gastrointestinal endoscopy identified oesophageal varices exclusively in patients with Caroli’s syndrome, including grade I varices in one patient and grade III varices in four patients. Four patients in the CS group experienced episodes of hematemesis during follow-up (at 9, 13, 17, and 20 months, respectively) and were successfully managed with endoscopic variceal band ligation.

During follow-up, a total of 94 medically supervised episodes of biliary cholangitis were documented across the study cohort. Management strategies varied according to clinical presentation and severity. Oral antibiotics were used in uncomplicated episodes, while broad-spectrum intravenous therapy was administered in episodes associated with systemic inflammatory features. Adjunctive therapies, including intravenous metronidazole and supportive measures such as ursodeoxycholic acid, spasmolytics, and antipyretics, were used as part of routine clinical care.

Patients were monitored longitudinally with periodic liver function tests and abdominal ultrasonography, as per the follow-up protocol. During follow-up, hepatic decompensation was documented in three patients (25%) within the Caroli’s syndrome group. These events occurred in the context of severe clinical episodes, including gastrointestinal bleeding and acute cholangitis. Biochemical evaluation demonstrated significantly higher serum total bilirubin and transaminase levels, along with significantly lower serum albumin levels, in patients with Caroli’s syndrome compared with those with Caroli’s disease, as summarized in Table 2.

During follow-up, one patient in the Caroli’s syndrome group developed left-sided pyelonephritis complicated by severe sepsis, which ultimately required nephrectomy. In addition, three patients with Caroli’s syndrome developed cholangiocarcinoma during follow-up, with a mean time to diagnosis of 28.3 ± 5.7 months from enrollment. These cases were characterized by progressive cholestasis and rising serum CA 19 − 9 levels. Endoscopic retrograde cholangiopancreatography was performed for biliary decompression, and metallic stents were placed for palliation. Due to poor clinical status, systemic chemotherapy was not administered, and management was limited to supportive and symptomatic care. All three patients died, with a mean survival of 3.3 ± 2.3 months following the diagnosis of cholangiocarcinoma.

An exploratory Spearman correlation analysis revealed several monotonic associations between clinical variables and adverse outcomes (Table 4). After applying Bonferroni correction within each outcome (adjusted α = 0.00625), portal hypertension, elevated CA 19 − 9 levels, and lower platelet count remained significantly correlated with increased cholangitis frequency. In contrast, none of the examined variables retained statistical significance for cholangiocarcinoma or all-cause mortality following correction for multiple testing. Associations observed for malignancy and mortality, including fever, hypoalbuminemia, thrombocytopenia, elevated bilirubin, INR, and CA 19 − 9, should therefore be interpreted as exploratory and hypothesis-generating. Given the limited sample size and low number of events, these correlations describe observed disease severity patterns rather than predictive relationships or causal effects.

**Table 4:** Exploratory spearman correlation analysis between clinical variables and adverse outcomes.

Variable	Cholangitis frequency (ρ, *p*)	Cholangiocarcinoma (ρ, *p*)	All-cause Mortality (ρ, *p*)
Fever (presence)	0.48, *p* = 0.041	0.52, *p* = 0.029	0.52, *p* = 0.029
Jaundice (presence)	0.46, *p* = 0.048	0.44, *p* = 0.057	0.44, *p* = 0.057
Platelet count	−0.61, *p* = 0.006	−0.58, *p* = 0.009	−0.58, *p* = 0.009
Serum albumin	−0.55, *p* = 0.015	−0.50, *p* = 0.026	−0.50, *p* = 0.026
Total bilirubin	0.53, *p* = 0.021	0.47, *p* = 0.038	0.47, *p* = 0.038
INR	0.57, *p* = 0.011	0.49, *p* = 0.031	0.49, *p* = 0.031
CA 19 − 9	0.62, *p* = 0.005	0.59, *p* = 0.008	0.59, *p* = 0.008
Portal hypertension	0.68, *p* < 0.001	0.55, *p* = 0.015	0.55, *p* = 0.015

**Fig. 1:**
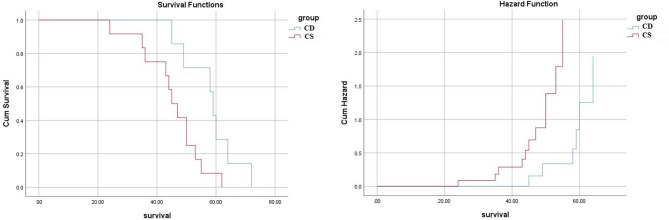
Kaplan–Meier survival curve showing higher survival in CD and CS groups.

**Fig. 2:**
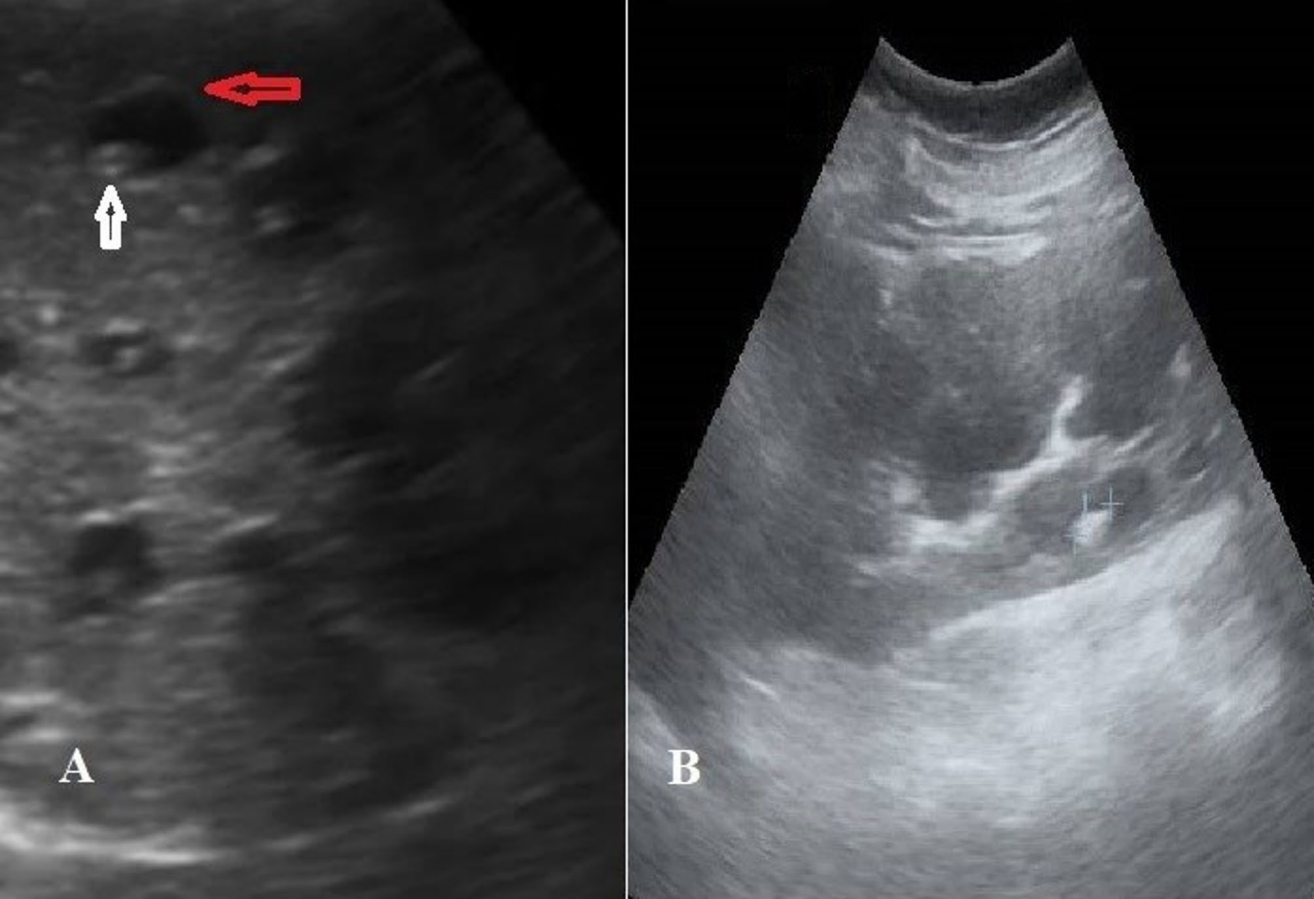
**A** Saccular dilation of intrahepatic ducts with central dot sign (white arrow) and surrounding dilated biliary radicles (red arrow). **B** Multiple renal cysts.

**Fig. 3:**
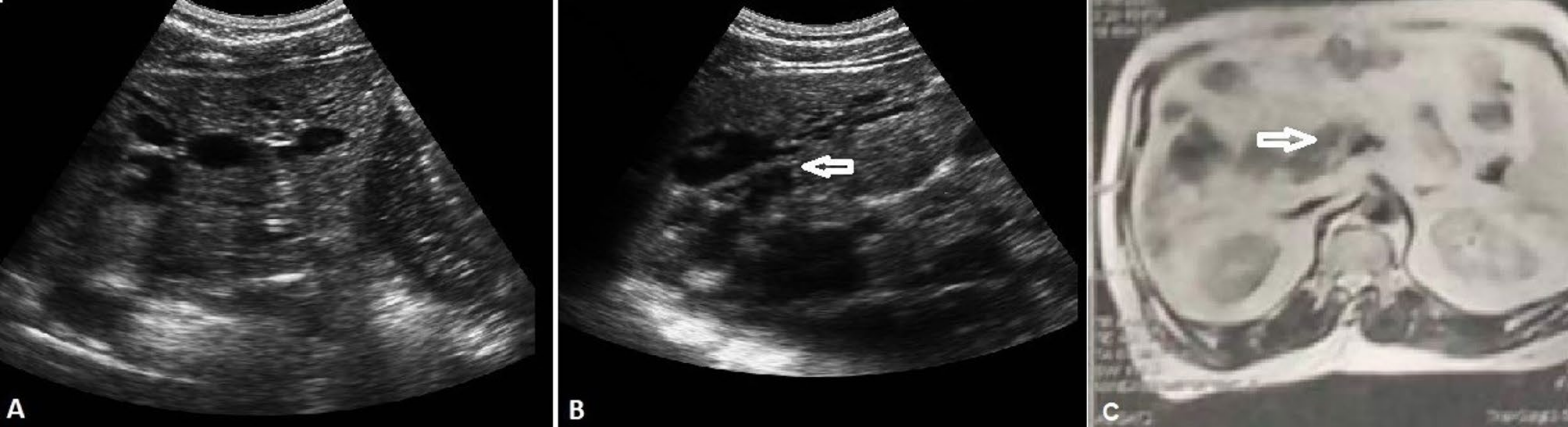
**A** Saccular dilation of intrahepatic ducts. **B** Saccular dilation of intrahepatic ducts, pancreatic cysts, and a hypoechoic mass with compressing effects (white arrow), causing dilation of intrahepatic biliary radicals. C: Triphasic CT confirming intrahepatic cholangiocarcinoma.

Survival patterns were examined using Kaplan–Meier estimates. Patients with Caroli’s disease (CD) had a mean survival time of 58.1 months (95% CI: 51.5–64.8) and a median survival of 59.0 months (95% CI: 56.4–61.6), reflecting the generally favorable outcomes observed in this group. Patients with Caroli’s syndrome (CS) had a mean survival of 45.3 months (95% CI: 39.6–51.1) and a median survival of 45.0 months (95% CI: 39.9–50.1). The log-rank test comparing the two groups yielded a p-value of 0.020. These findings are presented descriptively to summarize the observed longitudinal patterns and trends in survival; they should be interpreted as observational outcomes rather than evidence of causality or predictive associations, this plot shows also the Cumulative Hazard, representing the total accumulated risk of the event occurring up to a specific time: For both groups, the risk is negligible early on, CS climbs much steeper and earlier than CD, this confirms that the CS group faces a higher hazard (risk) rate throughout the observation period (Fig. 1). Non-overlapping or narrowly overlapping confidence intervals, especially for the medians, support a potentially meaningful difference in the survival distribution. This suggests that the observed difference in survival times, where CD had a median survival of 59 months, and CS had a median survival of 45 months, is unlikely to be due to chance (Figs. 2 and 3).

## Discussion

Caroli’s disease is a rare congenital disorder characterized by segmental, non-obstructive saccular dilatation of the intrahepatic bile ducts, generally arising from ductal plate malformation during embryonic development. When CD occurs in combination with congenital hepatic fibrosis and renal cystic disease, it is referred to as CS. Both conditions are considered part of the spectrum of fibropolycystic liver diseases and have been associated with mutations in the PKHD1 gene, which is also implicated in autosomal recessive polycystic kidney disease. Bile stasis within the dilated ducts can lead to recurrent cholangitis, intrahepatic stone formation, and a higher lifetime risk of cholangiocarcinoma. Given these potential complications, careful imaging surveillance and timely referral for surgical or liver transplant evaluation in progressive or complicated cases are recommended^13,14^.

The differential diagnosis of CD includes von Meyenburg complexes (biliary hamartomas), which typically appear as small, multiple, non-communicating cystic lesions. These lesions are often asymptomatic, incidentally identified on imaging, particularly magnetic resonance cholangiopancreatography and generally do not compromise liver function, although rare cases of malignant transformation have been reported. Other conditions in the differential diagnosis include polycystic liver disease and primary sclerosing cholangitis, both of which can present with cystic or biliary abnormalities on imaging^15,16^.

In the present cohort, three patients (25%) with Caroli’s syndrome developed cholangiocarcinoma. Given the small sample size, these observations should be interpreted descriptively rather than as definitive risk estimates. While previous literature reports the incidence of cholangiocarcinoma in Caroli’s disease to be up to 14%^17^, our findings reflect the clinical course within this limited cohort and underscore the potential for malignant transformation in CS. Both CD and CS are associated with congenital cystic dilation of the intrahepatic bile ducts, which may predispose patients to biliary tract malignancy. Mechanistically, factors such as chronic biliary stasis, recurrent inflammation from stones, and prolonged exposure of the biliary epithelium to bile constituents could contribute to epithelial injury and dysplasia. However, the present study is not powered to confirm causal pathways.

Furthermore, longstanding disease, aging, and inefficient bile drainage have been associated with a higher risk of neoplastic progression. Chronic inflammation resulting from recurrent cholangitis and fibrosis is thought to play a crucial role in activating oncogenic pathways in the biliary epithelium^18,19^. Although rare, mutations in oncogenes and tumor suppressor genes, such as KRAS and TP53, have been reported in cholangiocarcinomas arising in this context, further emphasising the neoplastic potential of this congenital biliary disorder. Diagnosing malignancy in patients with CS or CD remains challenging. The dilated and distorted biliary anatomy complicates detection, and current imaging tools often fail to identify early malignant changes. Consequently, routine screening for cholangiocarcinoma in these patients has had limited success.

In the present study, exploratory analyses indicated that several clinical and biochemical variables, including fever, severe thrombocytopenia, hypoalbuminemia, elevated total bilirubin, increased INR, elevated CA 19 − 9 levels, and portal hypertension, were nominally associated with cholangiocarcinoma development. However, after applying a Bonferroni correction to account for multiple comparisons, none of these variables remained statistically significant. Therefore, these findings should be interpreted as hypothesis-generating observations rather than definitive risk factors. They highlight potential markers that may guide future studies in larger cohorts but do not support prognostic decision-making in this small, rare-disease population.

Overall, while the incidence of cholangiocarcinoma in our CS cohort was higher than historically reported (25%), the small sample size and low event numbers underscore the need for cautious interpretation. These data reinforce the importance of careful longitudinal monitoring and support the descriptive, observational nature of our study, providing a foundation for future multicenter investigations that incorporate molecular and pathological analyses.

Previous studies have reported that cholangiocarcinoma in CS or CD tends to occur more frequently in older patients and those with long-standing disease, with reported incidences ranging from 2.7% to 37.5^20,21^. Elevated CA 19 − 9 levels, particularly ≥ 103 U/L, have been suggested as a potential marker of increased malignant risk and poorer outcomes in this population^22^. However, in the present study, while higher CA 19 − 9 levels were nominally associated with cholangiocarcinoma in exploratory analyses, this association did not remain statistically significant after correction for multiple comparisons. These observations underscore the limited ability of any single clinical or laboratory variable to reliably predict malignancy in small patient cohorts with rare diseases. Nevertheless, the combination of clinical features, biochemical markers, and longitudinal monitoring may still provide helpful guidance for identifying higher-risk individuals and tailoring surveillance strategies, laying the groundwork for hypothesis generation in future studies with larger cohorts.

In the current study, females accounted for 26.3% of patients in both the CS and CD groups, which is lower than the approximately 53% female representation reported in previous studies^21^. This discrepancy may reflect the small cohort size, referral patterns, or regional demographic differences. Liver failure occurred in three patients within the CS group, each following an episode of hematemesis related to portal hypertension, a complication well-documented in CS and consistent with prior observations^23,24^. These findings reinforce the more severe clinical phenotype of CS compared with isolated CD and highlight the need for careful monitoring of portal hypertension and its sequelae in this population.

The extent and severity of disease involvement should guide treatment strategies for patients with CD or CS. In patients with localized disease, particularly unilobar involvement, hepatic resection (e.g., segmentectomy or lobectomy) can provide symptom relief and may reduce the long-term risk of cholangiocarcinoma; retrospective studies report a post-resection malignancy rate of approximately 7%^25^. In contrast, for patients with diffuse or bilateral disease, especially those with portal hypertension, recurrent cholangitis, or progressive liver dysfunction, orthotopic liver transplantation remains the only definitive curative option and should be considered when resection is not feasible. These management principles underscore the importance of individualized treatment planning and ongoing surveillance to mitigate complications and optimize outcomes.

For patients who are not candidates for surgery or are awaiting liver transplantation, management remains primarily supportive and complication-focused. This includes prompt antibiotic therapy for cholangitis, endoscopic or percutaneous biliary drainage (ERCP or PTC) to relieve obstruction, endoscopic variceal ligation or nonselective beta-blockers to prevent variceal bleeding, and ursodeoxycholic acid to manage cholestasis and hepatolithiasis^26^. Several pharmacologic agents have been investigated for potential disease-modifying effects. Octreotide, a somatostatin analogue, has been proposed to reduce hepatic cyst volume and fibrosis by suppressing cyclic AMP-mediated cholangiocyte proliferation; however, evidence remains limited, mostly preclinical or anecdotal. Similarly, pioglitazone, a PPARγ agonist, has been hypothesized to mitigate bile duct dilation, fibrosis, and renal cystogenesis in fibropolycystic disorders. Still, clinical data on CD and CS are limited and extrapolated mainly from animal models or related conditions^27^. Overall, these approaches remain experimental, and management should prioritize prevention and prompt treatment of complications while individualized surveillance continues.

This study provides one of the most detailed clinical characterizations of Caroli’s disease and Caroli’s syndrome in a single-centre cohort over an extended follow-up period. Key strengths include the enrollment of a carefully selected group of patients with radiologically confirmed disease, enhancing diagnostic accuracy; a comprehensive diagnostic protocol including MRCP, extensive serological testing, and tumour marker evaluation allowing differentiation from mimicking conditions; and rigorous longitudinal follow-up (up to 7 years), enabling meaningful assessment of disease progression, complications (e.g., cholangitis, variceal bleeding, and cholangiocarcinoma), and survival outcomes. The study also describes associations between clinical and laboratory parameters (e.g., platelet count, CA19-9, INR, and frequency of cholangitis episodes) and adverse outcomes in patients with CS, providing hypothesis-generating insights that may guide future risk stratification and monitoring strategies in clinical practice.

However, the limitations that should be acknowledged include a small sample size due to the rarity of the condition and a single-centre, observational design. Exploratory correlations should be interpreted with caution: Given the limited number of events (e.g., deaths and cancer diagnoses), significant associations should be viewed as hypothesis-generating rather than confirmatory. Also, the absence of histopathological confirmation and genetic analysis represents a significant limitation. However, given the non-interventional nature of management and the rarity of surgical indications in this cohort, tissue sampling was not clinically justified. Future prospective studies incorporating molecular and pathological characterization are warranted.

The present study was not designed to identify prognostic or predictive factors. Instead, it provides a descriptive comparison of long-term clinical outcomes in CD and CS. Observed associations should be interpreted as exploratory and serve primarily to inform future, adequately powered studies.

In conclusion, CD generally follows a relatively benign clinical course, whereas CS is associated with a more severe phenotype, including higher rates of hepatic decompensation and biliary malignancy. These findings underscore the importance of careful clinical monitoring and proactive management of complications such as recurrent cholangitis, portal hypertension, and variceal bleeding, which may improve outcomes and delay liver failure. The study also describes associations between clinical and laboratory parameters and adverse outcomes, offering hypothesis-generating insights for risk assessment. Given the risk of cholangiocarcinoma, even among asymptomatic patients, regular clinical and imaging surveillance remains essential for early detection and timely intervention. Finally, while the small sample size and observational design limit the generalizability and preclude definitive predictive conclusions, this study establishes a foundation for multicentre and molecular studies that could further refine management strategies for this rare but clinically significant spectrum of fibropolycystic liver disease.

## Supplementary Information

Below is the link to the electronic supplementary material.

Supplementary Material 1

## Data Availability

Data from the present study can be obtained from the corresponding author upon reasonable request.
