# Supplementary material for: Clinical outcomes in Caroli disease and Caroli syndrome: a longitudinal observational cohort study
Source: Sci Rep. 2026 Mar 26;16:10482. doi: 10.1038/s41598-026-42855-8 (PMC13031803; doi:10.1038/s41598-026-42855-8)
Supplement: Supplementary file 1 — Supplementary Material 1 [file 41598_2026_42855_MOESM1_ESM.docx]

**Clinical Outcomes in Caroli Disease and Caroli Syndrome: A Longitudinal Observational Cohort Study.**

**Amr Shaaban Hanafy^1*^, Eslam Kamal Fahmy^2^, Rania Naguib^3^, Moaz Abulfaraj^4^, Ahmed F. Omar^5^, Hend Naguib^6^, Mohamed Mahmoud Abdelrahman^7,8^, and Hany A. Elkattawy^9, 10**^**

1. Internal Medicine Department, Gastroenterology and Hepatology Division, Zagazig University, Zagazig, Egypt; [amrhanafy@zu.edu.eg](mailto:amrhanafy@zu.edu.eg); ORCID ID 0000-0003-2901-2614
2. Department of Physiology, College of Medicine, Northern Border University (NBU), 91431, Arar, Saudi Arabia; [eslam.kamal.fahmy@gmail.com](mailto:eslam.kamal.fahmy@gmail.com); ORCID ID 0000-0002-1994-2570
3. Department of Internal Medicine, College of Medicine, Princess Nourah bint Abdulrahman University, P.O. Box 84428, Riyadh 11671, Saudi Arabia; [RNAbdalmoutaleb@pnu.edu.sa](mailto:RNAbdalmoutaleb@pnu.edu.sa); ORCID ID 0000-0003-2176-7182
4. Department of Surgery, Faculty of Medicine, King Abdulaziz University, Jeddah, Saudi Arabia; [maboalfaraj@kau.edu.sa](mailto:maboalfaraj@kau.edu.sa); ORCID ID 0000-0001-6893-537X
5. Department of Hepatology, Gastroenterology& Infectious Diseases, Faculty of Medicine, Zagazig University, Egypt; [farouqmail2000@yahoo.com](mailto:farouqmail2000@yahoo.com); ORCID ID 0000-0001-5716-2201
6. Internal Medicine Department, Hepatology Unit, Faculty of Medicine, Alexandria University, Alexandria, Egypt; [hendnaguib88@yahoo.com](mailto:hendnaguib88@yahoo.com); ORCID ID 0000-0001-6834-019X
7. Department of Anaesthesia and Critical Care, King Abdulaziz University Hospital, King Abdulaziz University, Jeddah, Saudi Arabia
8. Department of Anesthesia and surgical intensive care and pain management, Mansoura Faculty of Medicine. Mansoura University; [mabdelrahman@ksa.edu.sa](mailto:mabdelrahman@ksa.edu.sa); ORCID ID 0000-0002-4130-1729
9. Department of Basic Medical Sciences, College of Medicine, AlMaarefa University, Diriyah, 13713, Riyadh, Saudi Arabia; [hmohammed@um.edu.sa](mailto:hmohammed@um.edu.sa); ORCID ID 0009-0007-1778-1631
10. Research Centre, Deanship of Scientific Research and Post-Graduate Studies, AlMaarefa University, Diriyah, 13713, Riyadh, Saudi Arabia.

*** Running title:** Characteristics and Possible Outcomes in Caroli's Disease

*** Correspondence:** Hany A. Elkattawy, College of Medicine, AlMaarefa University, Diriyah, 13713, Riyadh, Saudi Arabia; [hmohammed@um.edu.sa](mailto:hmohammed@um.edu.sa); and Amr Shaaban Hanafy, Internal Medicine Department, Gastroenterology and Hepatology Division, Zagazig University, Zagazig, Egypt; amrhanafy@zu.edu.eg
